# At Your Fingertips: The Critical Role of Dorsalis Pedis Pulse Palpation in Early Detection of Peripheral Arterial Disease

**DOI:** 10.51894/001c.144459

**Published:** 2025-09-30

**Authors:** Esha Garg

**Affiliations:** 1 College of Osteopathic Medicine Michigan State University

## 80

### INTRODUCTION

Peripheral artery disease (PAD) is a common, yet potentially underdiagnosed, condition especially in those with diabetes or hypertension. The dorsalis pedis (DP) pulse, an important marker of lower extremity perfusion, can provide early clues of vascular compromise, as subtle changes may point to serious vascular issues. This case follows a 50-year-old woman with chronic foot pain who was initially thought to be neurovascularly intact. Her symptoms later revealed severe ischemic injury necessitating a below-the-knee amputation (BKA).

### CASE DESCRIPTION

A 50-year-old female with a history of type 2 diabetes, hypertension, pulmonary embolism and cerebrovascular accident with right-sided paresthesia presented to the emergency department with severe, chronic burning pain in her right foot, similar to her neuropathic pain. On examination, she was found to be neurovascularly intact in all extremities and was discharged with instructions to follow up with neurology. In a follow up clinic visit, she continued to report severe pain, and her pulses were noted to be good and equal in all extremities. She was given amitriptyline and pregabalin for better pain control. She returned to the emergency department with worsening symptoms, including a cool, purple right foot, absent DP and posterior tibialis pulses, decreased sensation, and reduced range of motion in her right lower extremity. Imaging revealed a thrombus in the abdominal aorta extending to the right common iliac artery, and she underwent emergency surgical embolectomy. Despite successful revascularization, her pain persisted, and she went through right BKA due to irreversible tissue damage.

### CONCLUSION/DISCUSSION

This case highlights the critical role of a thorough vascular exam in patients with chronic foot pain, especially those with risk factors like diabetes, previous thrombotic events and hypertension. The presence of an abdominal aortic thrombus resulted in a severe and atypical presentation of ischemic injury, leading to BKA. It emphasizes the necessity for careful and routine assessment of DP pulses to identify early signs of vascular pathology and prevent severe outcomes. Clinicians should remain alert of risk factors, perform and document a thorough vascular exam, and consider advanced imaging when symptoms are persistent, ensuring timely diagnosis and management of vascular emergencies.

